# A Review of the Treatments to Reduce Anti-Nutritional Factors and Fluidized Bed Drying of Pulses

**DOI:** 10.3390/foods14040681

**Published:** 2025-02-17

**Authors:** Shu Cheng, Timothy A. G. Langrish

**Affiliations:** Drying and Process Technology Research Group, School of Chemical and Biomolecular Engineering, The University of Sydney, Camperdown, NSW 2006, Australia; sche2348@uni.sydney.edu.au

**Keywords:** fluidized bed drying, anti-nutritional factors, pulses, exergy, energy consumption

## Abstract

Pulses, rich in proteins, dietary fibers, and essential nutrients, play an important role in human nutrition, especially as alternatives to animal proteins. However, the presence of anti-nutritional factors (ANFs), such as trypsin inhibitors, chymotrypsin inhibitors, phytic acid, and tannins, can hinder nutrient absorption, reduce protein digestibility, and impair the overall nutritional value of these foods (pulses). This literature review critically examines fluidized bed drying (FBD) as a promising method for processing pulses, with a focus on the effectiveness of FBD in reducing ANFs while preserving protein quality. The review highlights the impact of FBD on the quality and nutritional properties of pulses, discussing the effect of FBD on different kind of ANFs. Although FBD shows significant potential in reducing certain enzyme inhibitors, it has limitations in removing thermally stable ANFs, such as phytic acid. Furthermore, the review explores energy and exergy efficiencies in FBD systems, emphasizing the need for advanced technologies such as air recycle systems to enhance sustainability. This review identifies significant gaps in existing research, particularly in optimizing FBD methods for the effective removal of ANFs and in developing energy-efficient processing strategies.

## 1. Introduction

Pulses provide nutrients, such as proteins, dietary fibers, antioxidants, and resistant starches, that are needed for human health [1]. Pulses are also excellent meat protein substitutes. For example, the plant protein in plant-based meat comes from pulses [2]. However, some of these compounds are known to be “anti-nutritional factors” (ANFs) or “anti-nutritional compounds” (ANC). ANFs can reduce nutrient absorption, inhibit aspects of human metabolism, or affect protein digestibility. Therefore, ANFs are undesirable for human consumption [3]. It is essential to remove ANFs from pulses during processing using different treatments, such as soaking, cooking, dehulling, microwaving, and drying.

Fluidized bed drying (FBD) has been used in food processing due to its high thermal efficiency and relatively low operating costs [4]. Specifically, the application of FBD to pulses has been found to be effective in extending shelf life, reducing transportation costs, removing some ANFs, retaining key quality parameters, and maintaining nutritional values [5]. Current studies of pulse protein processing focus on nutrients and the quality of proteins. For example, the review by Mudryj et al. [6] reported that the saponins and tannins in pulses have antioxidant and anti-cancer effects. The protein, fiber, and vitamins provided in pulses are all important nutrients [6]. Shevkani et al. [7] studied the antioxidant properties of different types of pulses (including chickpeas, cowpeas, kidney beans, mung beans, lima beans, peas, and lentils) and the relationship between amino acid composition and antioxidants. For instance, chickpea-derived peptides, especially those rich in hydrophobic amino acids, have demonstrated significant antioxidant potential [7].

Preserving or enhancing the protein structure during drying is of great importance, as the integrity of the proteins is critical for maintaining the nutritional quality and functional properties of the final product. Therefore, optimizing drying parameters to minimize ANFs while safeguarding protein structure is essential for achieving both high product quality and efficient processing outcomes.

Moreover, fluidized bed drying is a widely utilized method in food processing that presents significant opportunities for optimizing energy and exergy efficiency. This review examines the advances in energy and exergy management within fluidized bed drying systems, particularly in the context of pulse processing. The energy demands of traditional open-loop systems are high, driven by substantial natural gas consumption for heating the fluidizing air [8]. Emerging technologies, such as fluidized bed dryers with air recycle, offer promising solutions by recycling hot air and thereby reducing energy consumption and processing costs [8]. Recent studies highlight that air recycle systems not only lower operational costs but also improve exergy efficiency and reduce carbon emissions [9]. A comparative analysis of different fluidized bed dryers, including widely used models, has not been carried out yet to consider the possibility that pressure drops, and thermal energy use may significantly impact exergy efficiency. This review provides an in-depth evaluation of these energy and exergy dynamics, emphasizing their implications for enhancing the sustainability and effectiveness of fluidized bed drying processes for pulses.

Finally, despite these advantages, significant challenges remain, particularly regarding the effective removal of thermally resilient ANFs, such as phytic acid and tannins. Although FBD is effective at reducing enzyme inhibitors, its ability to eliminate these thermally resilient (and frequently non-enzymatic) ANFs is limited, which can negatively affect the nutritional value and digestibility of pulses.

Previous literature review papers have surveyed the effects of various processing methods on ANFs, with a focus on dehulling, soaking, hydrothermal treatment, germination, fermentation, and irradiation [10,11,12]. However, there is a significant gap in this previous work in terms of the impact of drying processes. The perspective presented in this paper may contribute to the understanding of how drying can be employed to remove ANFs, as drying is an important step in pulse protein processing.

The objective of this review is to examine the effects of drying on ANFs. In Section 2 and Section 3, different types of ANFs will be analyzed. Section 4 will discuss the effects of drying on ANFs, while Section 5 will discuss modifications of the fluidized bed drying process for pulses, including improvements to the FBD system for greater energy efficiency and reduced costs. For instance, the energy-intensive nature of FBD, which requires high temperatures and significant gas flow rates, leads to high processing costs and raises concerns about sustainability. These challenges highlight the need for further research to optimize FBD systems, improve their efficiency in ANF removal, and develop methods for energy recovery and cost reduction.

## 2. Methodology

This literature review was conducted using a systematic approach to identify and analyze relevant studies on the effects of drying processes, particularly fluidized bed drying, on the reduction in anti-nutritional factors (ANFs) in pulses. The search strategy, inclusion criteria, and data extraction methods were carefully designed to ensure the comprehensiveness and reliability of the review. A structured literature search was performed using *Google Scholar* and *Web of Science*, two widely recognized academic databases. To capture a broad yet relevant dataset, the search was conducted using a combination of the following keywords: ANFs, pulses, fluidized bed drying, treatment, and exergy drop.

## 3. Pulse Proteins

Pulses are an important source of dietary protein and provide the body with a balanced supply of essential amino acids. The functional properties of pulse proteins are used in the development of various products, such as those from baking, noodles, and plant-based meat [13]. Nutritional imbalance due to the lack of protein is still a food problem faced by many countries. Pulses have the potential to become an alternative source of nutritional and functional proteins [14]. These proteins have multiple biological effects and provide sufficient essential amino acids and give good protein digestibility [15]. Technically, they provide suitable raw materials for the development of new products such as noodles, breads, and biscuits [15]. Although these pulses may be good sources of proteins, they contain a variety of ANFs that may cause nutritional limitations [16]. ANFs present in pulses are thought to impair protein digestibility and compromise the nutritional value of the pulses at high concentrations [17].

Table 1 shows the ANFs in the six major pulses. For example, according to Kumar’s review [10], trypsin inhibitor, chymotrypsin inhibitor, and phytic acid are three main ANFs for chickpeas. Trypsin and chymotrypsin inhibitors have high concentrations in chickpeas compared with the other five pulses, which are in a range (for chickpeas) of between 8.1 and 20.9 TIU/mg and 6.1–8.8 IU/mg, respectively [18]. The contents of saponins, haemagglutinin activity, and phytic acid are higher in faba beans than in chickpeas [10]. The content of phytic acid in faba beans can reach up to 98 mg/g [19]. For untreated faba beans, the saponin content is 137 mg/g [19]. The content of trypsin inhibitor in faba beans is relatively lower than in other pulses, such as chickpeas, mung beans, and lentils [20]. According to Table 1, the contents of ANFs in lentils, mung beans, and lupins are lower overall compared with the contents of trypsin inhibitor in chickpeas and phytic acid in faba beans. For field peas, the content of haemagglutinin can reach 80 HU/g, which is the highest content compared with the other five pulses [11]. Due to biological diversity, different types of pulses contain different types of ANFs, and the ANF contents vary significantly within each type of pulse.

## 4. Specific Anti-Nutritional Factors and Their Treatments

These antinutrients cause several biochemical pathways to be hindered. Pulses have been reported to have somewhat reduced digestibility compared with some protein sources because the presence of anti-nutrient enzymes involved in digestion reduces the bioavailability of nutrients [27]. ANFs in pulses can be divided into enzyme inhibitors, haemagglutinins, phytic acid, tannins, and saponins, based on their chemical and physical properties [28]. This section discusses the impact of different treatments on reducing the concentrations of ANFs.

### 4.1. Enzyme Inhibitor

An enzyme is a substance that speeds up certain biochemical reactions in the body, much like a catalyst does in a chemical reaction [29]. The main enzyme inhibitors in pulses include trypsin inhibitors, chymotrypsin inhibitors, and α-amylase inhibitors [20]. Both trypsin inhibitors and chymotrypsin inhibitors affect protein digestion in humans. α -amylase inhibitors are related to starch digestion [30]. Addressing the removal of these inhibitors is important for improving the digestibility and nutritional value of pulse-based products.

#### 4.1.1. Trypsin Inhibitor (TI) and Chymotrypsin Inhibitor

Trypsin inhibitors (TI) are defined as proteolytic enzymes that affect protein digestion [11]. Paraphrasing [11], “trypsin inhibitor is a group of serine protease enzymes. TI in pulses can be divided into Bowman-Birk trypsin inhibitors (BBTI) and Kunitz trypsin inhibitors (KTI) according to their molecular size”. The molecular weight of BBTI is smaller, at about 8 kDa, than KTI, which has a molecular weight of about 20 kDa [31]. Some pulses contain both types of trypsin inhibitors, such as soybeans, while lentils contain only one kind of TI [31]. The TIs of both have a negative effect on the biological activity of the digestive enzyme trypsin. Trypsin is called “trypsinogen” when it is in the pancreas and is present in an inactive form [32]. It is then activated upon entry into the small intestine to produce the trypsin enzyme. Trypsin inhibitors (TI) form irreversible complexes with trypsin [33], so trypsin levels in the intestines are reduced by the intake of TIs, resulting in reduced protein digestibility [34]. Improving the digestibility of pulse proteins by removing TI is helpful in pulses.

Table 2 shows different treatments to reduce TI activity in pulses. According to Nielsen et al. [35] and Subbulakshmi et al. [36], 6 to 10 days of germination can remove around 30–39% TI from pinto beans and French beans. Germination and soaking treatments can remove 30–40% of TI. Marquez et al. [37] tried different treatments to remove TI in chickpeas. Soaking overnight reduced 36% TI and boiling for 30 s removed 56%. TI activity increased with increasing boiling time [37]. After 300 s boiling, all the TI was removed. Another study from Cheng et al. [38] obtained similar results. Cheng et al. used fluidized bed drying to remove TI from chickpeas [38]. At the target moisture content, higher drying temperatures led to a greater reduction of TI. At the same time, the rate of TI removal was also related to the drying temperature. Overall, TIs are thermosensitive and can be effectively inactivated through thermal processing methods, such as boiling and fluidized bed drying. Thermal treatment facilitates the disruption of intermolecular bonds that maintain the tertiary structure of TIs, resulting in alterations to the conformation of their active sites [31].

The function of chymotrypsin inhibitors is similar to those of trypsin inhibitors, limiting protein digestibility. However, the site of activity for these inhibitors differs. While trypsin targets the amino acids, lysine and arginine, chymotrypsin specifically acts on hydrophobic residues such as tyrosine, tryptophan, and phenylalanine [12]. According to the results from Baintner [41], the removal of chymotrypsin inhibitors shows similar trends to trypsin inhibitor removal during heating processes for soybeans. The removal of chymotrypsin inhibitors required higher temperatures than that for trypsin inhibitor. Since fluidized bed drying involves thermal treatment, it presents a potential processing technique for effectively removing TI and CTI in pulses.

#### 4.1.2. α-Amylase Inhibitors

α-amylase inhibitors present in seeds can function as antinutritional factors for both human and animal nutrition [42]. These inhibitors reduce starch digestibility by inhibiting the activity of pancreatic and salivary α-amylase enzymes [30,43]. According to Table 3 from [12], the activity of α-amylase inhibitors is low in lentils, faba beans, and chickpeas. However, soybeans, black beans, pinto beans, and dark red kidney beans contain higher levels of α-amylase inhibitors, which is consistent with the results from [44]. Table 3 presents the changes in levels of α-amylase inhibitory activity in various types of beans. The results indicate that the soaking process does not significantly affect the α-amylase inhibitor activity in the pulses, as shown by the relatively small percentage reductions across the different types. In contrast, cooking results in a substantial reduction in α-amylase inhibitor activity for all pulse types, with reductions ranging from 80% to 100%. This suggests that cooking is far more effective than soaking in decreasing the α-amylase inhibitory activity in these beans.

According to Rekha and Padmaja, most thermal methods, including oven drying, cooking, and microwave baking, are effective in removing α-amylase inhibitors [45]. However, there is limited prior research that has thoroughly investigated the effectiveness of fluidized bed drying in removing these inhibitors from pulses. This represents a significant research gap.

### 4.2. Haemagglutinin

Haemagglutinins have been known to agglutinate red blood cells and impair various physiological and biochemical processes in mammals upon ingestion [46]. Table 4 shows the reduction of haemagglutinins under the different processing conditions. According to Bender [47], the amount and stability of haemagglutinin vary among different types of pulses. For example, whole yellow pea contains haemagglutinin, at 5.64 HU/mg dry matter [30]. Soybeans contain high levels of haemagglutinin, at 693 HU/mg dry matter [30]. More than 89% of haemagglutinin can be removed by cooking at 373 K for 2 min. After cooking for 20 min, 100% haemagglutinin can be removed from most varieties of pulses [47]. However, soaking does not significantly help to remove haemagglutinin, either from lentils [47] or from chickpeas [30]. According to Alajaji and El-Adawy, most kinds of heat treatment may effectively remove all of the HA from chickpea at high temperatures (over 373 K) [48]. However, Bender found that inadequate cooking can lead to food poisoning, as heating at 353 K for 15 min resulted in HA levels that were seven times higher than those in the raw beans. As the heating time increased, the HA content decreased correspondingly. Bender [47] also found that HAs are removed at 363 K. Overall, drying at high temperatures or for a long time is likely to remove HA.

### 4.3. Phytic Acid

**Figure 1:**
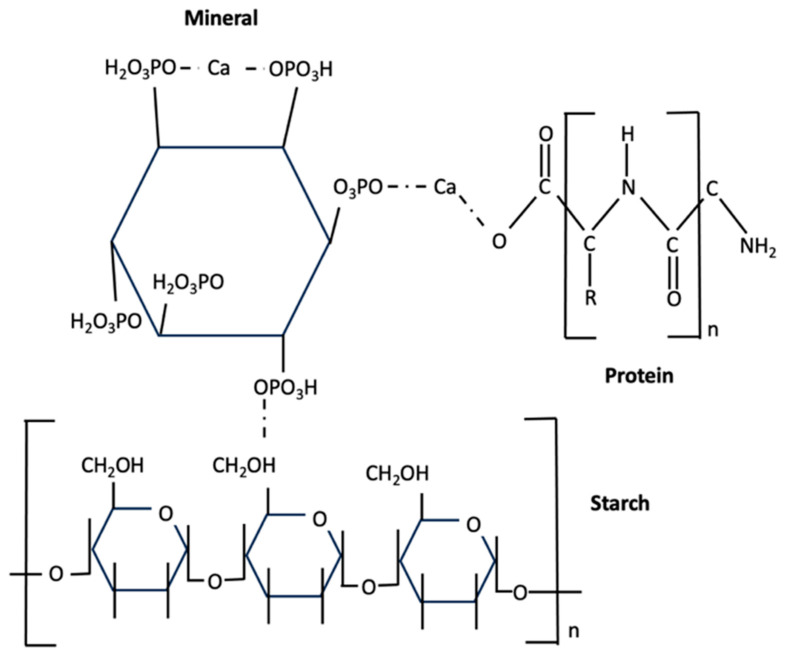
The complex between phytic acid and a mineral, a protein, and a starch. Adapted and with permission from Ref. [49]. Copyright 2025, Oatway et al.

Phytic acid is found in most grains, legumes, nuts, oilseeds, pipes, pollen, spores, and organic soil [49]. PA has been reported to bind (indirectly or directly) to minerals, proteins, and starches [49]. As shown in Figure 1, reactions occur between phytic acid and minerals, proteins, and starches. Due to the ability of phytic acid to bind to starch, it can reduce the conversion of starch into glucose. This may be helpful to a certain extent for low-glycemic index diets and diabetes control [50], but it may be undesirable at higher concentrations due to reduced availability of minerals in human digestion. The phosphate groups of phytic acid are negatively charged and can interact with positively charged components, such as many minerals and proteins. This combination affects the solubility, functionality, digestibility, and absorption processes for these minerals and proteins during digestion.

Paraphrasing [51], “for those who follow a balanced diet, phytic acid is not a health concern, but for those at risk for iron or zinc deficiency as well as vegetarians, phytic-rich foods may cause potential health risks”. This is particularly concerning in many developing countries, where whole grain cereals and legumes make up a large part of the diet [50]. There is a range of phytic acid concentrations in pulses, depending on the type of pulse and the processing involved [49]. Therefore, it is important to reduce the content of phytic acid during processing. Table 5 shows various amounts of reduction for phytic acid under different treatment conditions.

From Table 5, the reduction in phytic acid increased with microwaving time. At the same time, the temperature of the dehulled faba beans also increases with microwaving time. However, as the mass of faba beans increases, the reduction in phytic acid shows a downward trend at the same microwaving time.

The reduction in phytic acid during microwave processing is probably due to hydrolysis. Soaking before microwave treatment further enhances the potential for phytic acid hydrolysis. Phytic acid in dried grains is entirely in the form of a water-soluble salt. During processing, phytate is converted into pentaphosphate and tetraphosphate [56]. At the same time, high temperatures during microwaving may promote the hydrolysis reaction to a certain degree. Since phytic acid removal is caused by hydrolysis, boiling should remove phytic acid.

According to Alonso et al. [21], soaking and extrusion can reduce phytic acid levels by 30 ± 3%. Germination for 72 h can remove phytic acid concentrations by 59%. However, Shi et al. [30] found that there was no detectable decrease in phytic acid levels after soaking for any pulses. Table 6 shows that environmental factors may affect the removal of phytic acid for different types of pulses under different soaking conditions (different temperatures, different pH values) [16,57,58]. Germination is an effective method for removing phytic acid. The amount of phytic acid removed is proportional to the duration of germination. Alonso et al. found that germination for 24, 48, and 72 h removed 54, 59, and 61% of phytic acid, respectively [21]. In addition, microwaving is a potential method to remove phytic acid. According to Sharif et al. [59] and Manez et al. [60], soaking and microwaving the pulses together can remove 49 ± 4% of the phytic acid content. Daneluti et al. [61] found that phytic acid thermally decomposes at a temperature of 380 °C, resulting in the reduction of carbon and hydrogen. This situation means that phytic acid is thermally stable during this microwaving, and increasing the temperature may not be the optimal way to remove phytic acid. The study by Cheng et al. found consistent results, indicating that increasing the temperature is not an optimal method for removing phytic acid [62]. As a result, fluidized bed drying did not lead to a significant reduction in phytic acid concentration. In contrast, soaking reduced phytic acid levels to a certain extent, while microwaving had a more pronounced effect in reducing phytic acid levels in faba beans.

### 4.4. Tannins

Tannins are one of the most common antinutritional factors. They are present not only in pulses but also in most plants [64]. Tannins are defined as complex, amorphous, and water-soluble polymeric phenolic substances [65,66]. Tannins can be divided into three major types: hydrolysable tannins, phlorotannins, and condensed tannins [64,67]. Phlorotannins are the simplest tannin group in terms of their structures [67] and can be detected in aquatic species such as brown algae [68]. Condensed tannins are more widely distributed than hydrolysable tannins. Tannins can precipitate proteins [64,67]. This property of tannins is used to transform raw animal into leather [69], because tannin molecules cross-link proteins, making these proteins more resistant to bacterial and fungal attacks [67]. Tannins, on the other hand, may reduce the activity of many enzymes [67]. One of the main characteristics of tannins as digestive inhibitors or toxins in humans or animals is that they form chemical complexes with substances such as proteins, polysaccharides, alkaloids, nucleic acids, steroids, and saponins, reducing the ability of the human body to absorb these nutrients [70]. Therefore, removing tannins from pulses is an important part of food processing for these materials.

The characteristics and amounts of tannins in pulses are affected by processing due to their high reactivity [66]. Alonso et al. [21] and Khandelwal et al. [66] have shown that germination can remove over 50% of any tannins present. The germination process in the work of Khandelwal et al. [66]. was performed by soaking the pulses in tap water overnight at room temperature for 12 h, then wrapping the sample pulses in a moist cloth and leaving them at room temperature for 24 h. For lentils, after germination, the tannins could not be measured, suggesting that they are entirely removed by germination [66]. According to the results from Table 7, boiling for 90 min can remove 29% of tannins from lentils [48]. With increased boiling time, for example, boiling faba beans until they are soft, 76% of tannins may be removed [27]. Cooking under high pressures has been reported to improve the removal of the tannins [48]. Microwaving has also been reported to remove half of the tannin content in lentils [22]. Dehulling for faba beans has been reported to remove about 92% of the tannins [21]. Overall, most treatments, including cooking, microwaving, boiling, dehulling, germination, and extrusion are helpful for removing tannins. Relating to cooking and boiling, tannin reduction increases with an increase in the cooking temperature or pressure. Dehulling and germination are also both effective at removing tannins.

## 5. Fluidized Bed Drying Technologies for Pulses, and the Effects of Drying on Anti-Nutritional Factors

The fluidized bed drying of pulses has been studied by several researchers, such as Darvishi, Prachayawarakorn, and Dondee, who have focused on different ways to dry pulses in fluidized beds [71,72,73]. For pulses, there are at least five common physical quality characteristics, such as cracking and breakage [71,72], color testing by color meter [71], protein solubility [71,72,73], the concentration of ANFs [38,73,74], and moisture content [71,73,74]. According to the study from Darvishi et al. [71], fluidized drying at high temperatures can cause V-shaped cracks in pulses. During the drying process, the movement of water is limited by water diffusion in pulses, and the diffusion rate of water increases as the temperature rises. The drying of the surfaces causes the surface temperature of pulses to rise above the wet-bulb temperature. Therefore, at higher drying temperatures, the surfaces of the kernels become brittle and prone to cracking. Dondee et al. [72] showed that fluidized bed drying resulted in minor changes in the color of pulses. From the results of Cheng and Langrish [38], the fluidized-bed drying process may help reduce the concentration of ANFs, specifically trypsin inhibitors in chickpeas.

The fluidized bed drying of pulses is relevant to reducing some ANFs in pulses and grains. As shown in Table 8, the fluidized bed drying process can remove some ANFs. According to the results of Osella et al. [40], the rate of trypsin inhibitor inactivation increases with higher fluidized bed drying temperatures, a result also reported by Cheng et al. [38]. This study [38] further demonstrated that using a drying schedule removed 86% of the trypsin inhibitor at the target moisture content, and the schedule also reduced the denaturation of the protein secondary structure compared with drying at a constant temperature and humidity [38].

For α-amylase inhibitors, drying at 363 K for two hours removed 80% of these inhibitors [45]. This suggests that fluidized bed drying is effective in removing enzyme inhibitors, such as trypsin inhibitors and α-amylase inhibitors. However, HAs are slightly different from enzyme inhibitors and cannot be removed at low temperatures or short drying times, and higher temperatures (over 363 K) or long drying times are needed to remove them [47].

For thermally stable ANFs, such as phytic acid and tannins (non-enzymatic inhibitors), the fluidized bed drying process has limitations. Pande et al. [75] found that fluidized bed drying of green gram seeds at 323 to 343 K slightly reduced the phytic acid content but did not completely remove it. The study by Cheng et al. [62] gave the same results with a fluidized bed dryer and found that microwaving before fluidized bed drying could effectively remove phytic acid and partially dry the pulses. The study by Muetzal and Becker indicated that tannin activity was not affected by the drying method [76]. Boiling and cooking for short processing times do not appear to be effective, but microwaving and germination may be a reasonable way to remove tannins.

Overall, fluidized bed drying is a promising method for reducing certain enzyme inhibitors in pulses, such as trypsin and α-amylase inhibitors. However, its effectiveness is limited when dealing with thermally stable ANFs, such as phytic acid and tannins. To overcome these limitations, combined microwave and fluidized bed drying may be attempted in future work.

## 6. Modifications of the Fluidized Bed Drying Process in Pulses

Fluidized bed drying is a widely utilized technology in the pharmaceutical and food industries for solids processing. This technique offers high heat and mass transfer coefficients between solid particles and the drying medium, enabling efficient moisture removal under relatively mild conditions [38]. Therefore, fluidized bed drying minimizes product degradation, making it a promising approach for the processing of pulse proteins.

The working principle of a fluidized bed is that a fluid (e.g., air here) passes up through a bed of granular solids, such as pulses. At low fluid velocities, the bed of solids remains stationary, which is called a fixed or packed bed. At higher fluid velocities, the bed is lifted by the fluid flow, and the granular solids move freely, resulting in thorough solids mixing and good product uniformity, which is essential to give good product quality and a uniform moisture content.

However, there are two major problems for the fluidized bed drying of pulses. Firstly, the process is unable to eliminate all types of ANFs, such as non-enzymatic inhibitors, including phytic acid and tannins. Secondly, the fluidized bed drying process is associated with high energy consumption and processing costs. This is primarily due to the necessity to maintain high temperatures and gas flow rates to achieve the desired drying efficiency, which further challenges the economic feasibility of the process. Consequently, these limitations necessitate ongoing research and development to optimize the ANFs removal process and enhance energy reuse and recovery during the fluidized bed drying process for pulses.

### 6.1. Combined Microwave and Fluidized Bed Drying (CMFD)

**Figure 2:**
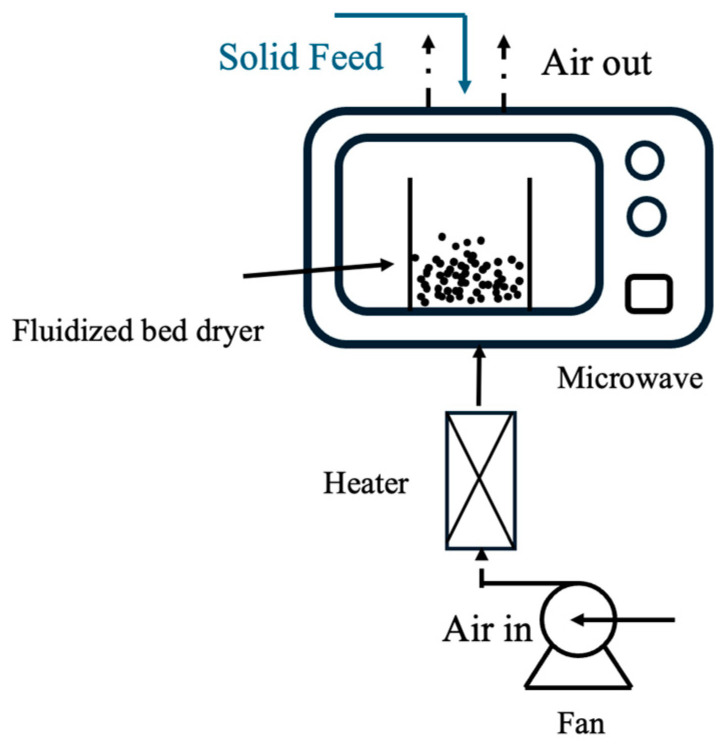
Schematic diagram of a CMFD system showing the gas flow direction (the air is supplied by the fan and heated by the heater).

For the first challenge, several studies have highlighted the effectiveness of microwaving in removing thermally stable ANFs, such as phytic acid [62,77] and tannins [77], in a short period of time. For example, Sharma et al. report that microwaving for 40–100 s could remove up to 77% tannins from sorghum [77]. Similarly, a previous study by Cheng et al. found that microwaving for two minutes could effectively remove most of the phytic acid from faba beans [62]. However, compared with fluidized bed drying, microwaving is less effective in removing trypsin inhibitors. For example, Sharma et al. found that microwaving 30 min removed only 12–13% of trypsin inhibitors from buckwheat [77]. Therefore, CMFD could be a promising process for effectively reducing a wide range of ANFs in pulses. The schematic diagram of a CMFD system with gas flow direction shown in Figure 2.

In previous studies, CMFD has been applied in grain processing, including peppercorns [78], carrots [79], soybeans [80], and brown rice [81]. As outlined in Table 9, CMFD significantly accelerates the drying rate of fresh peppercorns, leading to a more effective drying process [78]. Additionally, CMFD has proven to be effective in drying diced carrots, resulting in improved drying efficiency and uniform moisture content [79]. According to Khoshtaghaza et al., CMFD optimizes the quality of soybean kernels while simultaneously reducing energy consumption and improving drying kinetics [79]. Chupawa et al. demonstrated that the CMFD approach, combined with stepwise microwave heating and bed height control, improves the performance in preparing instant brown rice [80]. Similarly, Zare and Ranjbaran found through performing simulations that CMFD effectively achieves uniform drying in soybeans, coupled with enhanced drying rates [81].

In summary, while CMFD has demonstrated its effectiveness in enhancing drying performance in grain processing, its application for the removal of ANFs from pulses has not been extensively studied. The literature review indicates that CMFD holds considerable promise as a technique for reducing a broad spectrum of ANFs in pulses. However, the scarcity of research in this specific area underscores a significant gap, warranting further investigation to fully leverage the potential of CMFD in improving the nutritional quality of pulses.

### 6.2. Energy and Exergy Saving During Fluidized Bed Drying

Drying is a process with substantial energy demands, necessitating the study of potential reductions in energy and exergy consumption [8,9]. The adoption of emerging technologies is critical for advancing energy efficiency in this area.

#### 6.2.1. Air Recycle Fluidized Bed Drying: Energy and Cost Saving

**Figure 3:**
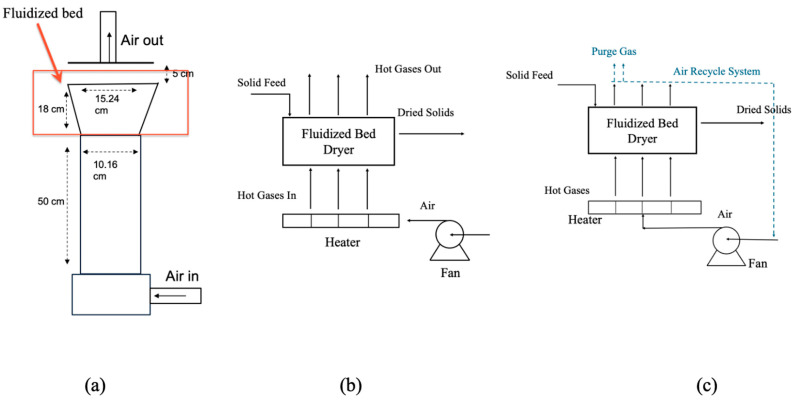
Schematic diagram of a fluidized bed drying system. (**a**) The fluidized bed used in the study of Cheng and Langrish [38], adapted with permission from Ref. [38], copyright 2025, Shu Cheng and Timothy Langrish. (**b**) Schematic diagram of an open-loop fluidized bed drying system with gas flow direction. (**c**) Schematic diagram of an air-recycle fluidized bed drying system showing the gas flow direction (the air is supplied by the fan and heated by the heater).

The operating costs of an open-loop, straight-through airflow system are very high, primarily due to the substantial energy expenses required to heat the fluidizing air. Consequently, incorporating some degree of air recycling emerges as a potential strategy for reducing energy consumption. As illustrated in Figure 3b, an air recycle fluidized bed dryer effectively recycles hot air during the drying process, thereby reducing processing costs. Moejes et al. demonstrated that an air recycle dryer system can significantly decreases the energy consumption during milk powder production [9]. Furthermore, Lei and Langrish emphasized that closed-loop systems, where air recycle is similar to a closed-loop, not only enhance exergy efficiency but also contribute to a reduction in carbon dioxide emissions [8]. Exergy is energy availability, which is a measure of energy quality, and exergy will be defined in the following section. Overall, air-recycle drying systems offer a good alternative to conventional methods due to their numerous potential advantages [83].

#### 6.2.2. Exergy Analysis and Pressure Drop

A distinctive feature of a fluidized bed dryer from energy and exergy perspectives, apart from the use of thermal energy for drying, is the pressure drop across the distributor and the pressure drop involved in supplying the drying air to the equipment. This section compares the exergy consumption between a commercial small-scale fluidized bed dryer (14 cm × 12 cm × 6.6 cm) that uses compressed air for the main air flow, and the fluidized bed dryer used in the study by Cheng and Langrish [38,62]. The commercial dryer is a widely used small-scale fluidized bed dryer and granulator, particularly in the pharmaceutical and catalysis industries. For instance, Chen et al. employed this type of equipment in the development of a pharmaceutical powder [84], while Leung et al. used this type of equipment to enhance the drying of catalysts [85]. The principal feature of the commercial fluidized-bed dryer, for the purpose of this analysis, is the use of compressed air at 6 bar to create the main air flow through the fluidized bed equipment. For comparison, the fluidized bed dryer used in Cheng and Langrish is shown in Figure 3a [38].

A definition for exergy that includes both temperature and pressure components (of exergy) may be given as follows [8,86]:

(1) Ex=m˙CpTin−Tout−Tout ln⁡TinTout+Toutln⁡pinpout

where $\dot{m}$ is the mass flow rate for the stream (kg/s), *T_in_* and *T_out_* are the inlet and outlet stream temperatures (K), respectively, *p_in_* and *p_out_* are the inlet and outlet stream pressures (Pa), respectively, and *C_p_* is the specific heat capacity for the stream (around 1000 J/(kg K) for air).

To specify a case study, suppose that the inlet temperature and pressure to the fluidized bed are 353 K and 101,500 Pa (just above standard atmospheric pressure, accounting for the pressure drop across the fluidized bed), respectively. Table 10 shows the inlet and outlet stream temperatures in the commercial dryer and the fluidized bed dryer used in the study by Cheng and Langrish [38]. The temperatures in the commercial dryer were obtained from Chen et al.’s study [84].

The pressure drop across the fluidized bed dryer in the study of Cheng and Langrish was measured to be less than 50 Pa [38], and the exergy drop across the fluidized bed due to this pressure change is given by the following calculation:

(2) $$\frac{Ex}{\dot{m}}=1000\;J\;{kg}^{-1}K^{-1}\;307\;K\ln\left[\frac{\left(101,500\;\right)\;Pa}{\left(101,500-50\right)\;Pa}\right]=151\;J\;{kg}^{-1}$$

This exergy drop across the bed due to the pressure drop may be compared with the corresponding exergy drop for typical temperature drops across the fluidized bed dryer using the same equipment, as reported by Cheng and Langrish [38]. When the inlet air temperature of the fluidized dryer is 353 K, the temperature of the outlet air is 303 K. The temperature drop across the fluidized bed dryer is 50 K, and the exergy change across the bed is given by the following calculation:

(3) $$\frac{Ex}{\dot{m}}=1000\;J\;{kg}^{-1}K^{-1}\;\left[\left(353\;K-303\;K\right)-303\;K\ln\left(\frac{353\;K}{303\;K}\right)\right]=3721\;J\;{kg}^{-1}$$

The pressure drop of 50 Pa across the fluidized bed has a lower exergy drop (151 J kg^−1^) than a temperature drop of 50 K, so the loss in exergy due to pressure drops in this fluidized-bed system is negligible compared with the loss in exergy due to thermal energy changes (3721 J kg^−1^).

However, this negligible exergy drop due to the bed pressure drop is not always the case for fluidized bed dryers. For example, a typical commercial fluidized bed dryer in the pharmaceutical industry [85] involves reducing the pressure of compressed air from at least 6 bar to atmospheric pressure in order to supply the drying air to the equipment. The corresponding exergy drop for this pressure drop is given by the following calculation:

(4) $$\frac{Ex}{\dot{m}}=1000\;J\;{kg}^{-1}K^{-1}\;300\;K\ln\left(\frac{600,000\;Pa}{101,325\;Pa}\right)=533,579\;J\;{kg}^{-1}$$

This exergy drop would correspond to a temperature drop of 46 K, as shown by the following calculation:

(5) $$\frac{Ex}{\dot{m}}=1000\;J\;{kg}^{-1}K^{-1}\;\left[\left(353\;K-307\;K\right)-307\;K\ln\left(\frac{353\;K}{307\;K}\right)\right]=3137\;J\;{kg}^{-1}$$

To achieve an exergy drop of 533,579 J/kg due to a pressure drop of 6 bar, the corresponding temperature drop must exceed 540 K. Consequently, when compressed air at 6 bar is utilized as the fluidizing gas in a commercial fluidized bed dryer, the exergy drop attributed to the pressure drop is significantly larger than that caused by temperature changes. As shown in Table 11, the commercial equipment is therefore not as exergy efficient as the fluidized bed dryer used in the study of Cheng and Langrish [38].

Overall, both temperature difference and pressure drop are important in fluidized bed drying. Temperature difference is an inevitable consequence of drying, and it is always present, being proportional to the drying rate. However, the extent of pressure drop depends on the design of the dryer. The calculations here show that the design of the air distributor for the fluidized bed, which is critical to the exergy loss due to pressure drop, is a controllable parameter in the design process for a fluidized bed dryer. The exergy loss due to the pressure drop over the air distributor may be the dominant parameter in a fluidized bed where the design is sub optimal, but in a well-designed fluidized bed, it should be a smaller term and comparable to the exergy drop due to drying and the accompanying temperature difference.

## 7. Conclusions

Fluidized bed drying (FBD) is an effective method for enhancing the quality of pulses; however, it has certain limitations, particularly in the removal of anti-nutritional factors (ANFs). This review examined the impact of FBD on various ANFs, including enzyme inhibitors, phytic acid, haemagglutinin, and tannins. Among these, enzyme inhibitors are readily degraded through FBD, while haemagglutinin requires higher drying temperatures and prolonged drying times for effective removal. Conversely, thermally stable ANFs such as phytic acid and tannins exhibit resistance to conventional FBD. To address this challenge, hydrolysis-based treatments are often employed, necessitating prior soaking or wetting of pulses to facilitate enzymatic or chemical reactions. However, to achieve the desired low moisture content for storage stability, a subsequent drying process is required. Another notable limitation of FBD is the significant exergy drop, which affects energy efficiency.

To mitigate these challenges, this review explores potential solutions. The combined microwave-fluidized bed drying (CMFD) process presents a promising approach to effectively reduce a wide range of ANFs while preserving pulse quality. By integrating hydrolysis with drying, CMFD enhances processing efficiency. The application of microwaves accelerates drying rates, thereby improving overall process performance. Additionally, to minimize exergy loss during FBD, optimizing both temperature differentials and pressure drops is crucial. This review highlights that while temperature variations are inherent to the drying process, a well-designed fluidized bed system can effectively reduce pressure drop, thereby improving energy efficiency. Future research should focus on optimizing CMFD parameters and exploring innovative designs to enhance the effectiveness and sustainability of pulse processing technologies.

## Figures and Tables

**Table 1:** The main anti-nutritional factors in chickpeas, faba bean, lentils, mung beans, lupins, and field peas. Adapted from Ref. [10].

Pulses	Trypsin Inhibitors (TIU/mg Protein)	Chymotrypsin Inhibitor (IU/mg)	α-Amylase Inhibitor (IU/g)	Haemagglutinin (HU/g)	Phytic Acid (mg/g)	Oxalate (mg/100 g)	Tannins (mg/g)	Saponins (mg/g)	References
Chickpeas	8.1–21	6.1–8.8	3.1–11	6.2	5.8–12	70.3	0.04–4.9		[11,18]
Faba beans	2.3–7.2	3.6	19	49	32–98	194	0.3–21	31–137	[11,19,21]
Lentils	3.6–7.6	0	0	50	4.1–13	13.3	1.3–3.9	180–1595	[11,22]
Mung beans	16			27	5.8–12		3.3		[23,24]
Lupins	≤0.1				0.41–14				[25,26]
Field peas	0.8–6.3	2.7–4.9	17	80	3.0–13		2.8–3.1		[11,22]

**Table 2:** Activity reduction for trypsin inhibitors in pulses under different treatment conditions.

Pulses	Processing Conditions	Trypsin Inhibitor Activity Reduction (%)	References
French beans	10 days germination	30	[35]
Pinto beans	6 days germination	39	
Chickpeas	Soaked in water at room temperature for 16 h	36	[36]
Chickpeas	Boiled in water at 96 °C, 30 s	56	[37]
Chickpeas	Boiled in water at 96 °C, 300 s	100	[37]
Soybeans	Autoclaved at 121 °C, 60 min	96	[39]
Soybeans	Fluidized bed dried at 120 °C, 10 min	85	[40]
Chickpeas	Fluidized bed dried drying schedule 2 h	86	[38]

**Table 3:** Activity reduction for α-amylase inhibitors in pulses under different treatment conditions.

Pulses	Processing Conditions	α-Amylase Inhibitor Activity Reduction (%)	Reference
Dark red kidney bean	Soaked in room temperature, 4 h	11	[12]
Soybean		4	
Black bean		7	
Pinto bean	Boiled in 95 °C, 1 h	80	
Navy bean		93	
Dark red kidney bean		90	
Soybean		100	
Black bean		91	
Sweet potato flour	Oven dried in 90 °C, 2 h	80	[45]
Taro tubers	Cooked for 30 min	86–88	
Sweet potato flour	Microwaved 120 s	65–100	
Taro tubers	Microwaved 120 s	63–91	

**Table 4:** Activity reduction for haemagglutinin (HA) in pulses under different treatment conditions.

Pulses	Processing Conditions	Haemagglutinin Activity Reduction (%)	References
Red lentils	Soaked overnight	22	[47]
Red lentils	Cooked at 373 K for 2 min	97	[47]
Red lentils	Cooked at 373 K for 20 min	100	[47]
Chickpeas	Soaked	1.7	[30]
Chickpeas	Cooked	94	[30]
Chickpea	Autoclaved	100	[48]
Chickpea	Microwaved	100	[48]

**Table 5:** Microwave processing to reduce phytic acid and the reasons.

Microwaving	Reduction in Phytic Acid	Reasons	Reference	Years
900 W, 2450 MHz, 2–12 min	34.7–35.6%	Low levels of inositol and inositol phosphate due to the effects of free radicals produced during irradiation	[52]	2012
2450 MHz, 15 min	17.6–54.4%	Decreased water extractability of phytate caused by heating processes which included pressure cooking, microwave cooking, and roasting	[53]	2016
2450 MHz, 30 min	62.69–64.88%	Phytic acid is relatively heat-labile, hence it was decreased very easily after thermal processing, especially autoclaving	[54]	2018
2450 MHz, 30 min	24.6%	Hydrolysis during soaking, sensitivity to thermal treatment, and insoluble complexes formed between phytic acid and other components	[55]	2015

**Table 6:** Phytic acid reduction in pulses under different treatment conditions.

Pulse	Processing Conditions	Phytic Acid Reduction (%)	References
Faba beans	Soaked	32.7	[21]
Faba beans	Germination for 72 h	61	[21]
Goat peas	Soaked at room temperature	3.9	[57]
Lentils	Soaked under acidic condition	37	[16]
India tribal pulse	Soaked under alkaline condition	11	[63]
Lentils	Microwaved + soaked	45–52	[59]
Vicia faba	Extrusion	27	[21]
Lentils	Microwaved + soaked	45	[60]
Faba beans	Fluidized bed dried at 120 °C for 15 min	15	[62]
Faba beans	Fluidized bed dried at 140 °C for 15 min	22	[62]
Faba beans	Soaked overnight + Microwaved 2 min	100	[62]

**Table 7:** Tannin reductions in pulses under different treatment conditions.

Pulses	Processing Condition	Tannins Reduction (%)	References
Chickpeas	Pressure-cooked (15 psi) at 394 K, 35 min	84	[48]
Chickpeas	Microwaved	48	[48]
Lentils	Boiled at 373 K, 90 min	29	[22]
Lentils	Pressure cooked at 394 K,35 min	36	[22]
Faba beans	Boiled in water at 373 K, until soft	76	[19]
Faba beans	Dehulled	92	[21]
Faba beans	Germinated 24 h	56	[21]
Faba beans	Extrusion	54	[21]
Green grams	Germinated for 24 h	54	[68]
Lentils	Germinated for 24 h	100	[68]

**Table 8:** The removal of different ANFs by fluidized bed drying.

Drying Conditions	Pulses/Grains	ANFs	Finds	References
Drying at 120 °C for 10 min	Soybeans	Trypsin inhibitor	Reduce 90% trypsin inhibitors	[40]
Drying schedule (353 K for first 30 min and 333 K for 90 min)	Chickpeas	Trypsin inhibitor	Reduce 90% trypsin inhibitors	[38]
Dried in 363 K for 2 h	Sweet potato flour	α-amylase inhibitors	Reduce 80% α-amylase inhibitors	[45]
Drying temperature from 323 K to 343 K	Green gram seed	Phytic acid	No significant effects, in the range of 600–630 mg/100 g	[75]
Drying temperature from 393 K to 413 K	Faba beans	Phytic acid	No significant effects	[62]

**Table 9:** Summary of studies on the efficacy of combined microwave/fluidized bed drying (CMFD) in grain processing.

Material	Processing	Findings	Reference
Peppercorns	CMFD	Reduces 85 ± 5% drying time, enhances color, and preserves peppercorn structure	[82]
Carrot	CMFD	CMFD dryer is 2–5 times faster than a fluidized bed dryer	[78]
Soybean	CMFD	Drying time can be shortened by 30–150 times by using CMFD, energy saving	[79]
Brown rice	CMFD	Lower microwave power reduces charring, minimizing color change in drying	[80]
Soybean	CMFD	Uniform drying, enhance drying rate	[81]

**Table 10:** Inlet and outlet stream temperatures in the two different fluidized bed dryers.

Type	*T_in_* (K)	*T_out_* (K)	Δ*T* (K)
Commercial dryer	353	303	50
Fluidized bed used in Cheng and Langrish’s study	353	307	46

**Table 11:** Summary of the exergy drop and temperature drop between the fluidized bed dryer used in Cheng and Langrish’s study [38] and a commercial fluidized bed dryer.

	Fluidized Bed Dryer Used in Cheng and Langrish’s Study (Figure 3a)	Commercial Fluidized Bed Dryer
Pressure drop (Pa)	50	600,000
Exergy drop due to pressure drop (J kg^−1^)	151	533,519
Difference between inlet and outlet temperatures (K)	50	46
Exergy drop due to temperature drop (J kg^−1^)	3721	3137

## Data Availability

No new data were created or analyzed in this study.
